# Molecular Identification and Pathogenicity of *Fusarium* Fungi Causing Potato Dry Rot in Shanxi Province, China

**DOI:** 10.3390/jof11120835

**Published:** 2025-11-25

**Authors:** Jiaru Guo, Yupei Shi, Xi Chen, Peibing Du, Yingli Zhao, Liang Wang

**Affiliations:** 1 College of Food Science and Engineering, Shanxi Agricultural University, Taiyuan 030031, China; jiaruguo0818@163.com (J.G.); shiyupei6163@163.com (Y.S.); chenxi7441@163.com (X.C.); zhaoyingl@hotmail.com (Y.Z.); 2 High Latitude Crops Institute, Shanxi Agricultural University, Datong 037008, China; dupeibing@126.com

**Keywords:** potatoes, *Fusarium* spp., multilocus sequence analysis, postharvest disease

## Abstract

In the present study, 70 representative strains of potato dry-rot pathogen fungi were collected and isolated from three potato-growing areas in Northern Shanxi Province to determine their distribution and composition. The aim was to determine their distribution and composition by investigating their genetic structure through morphological characterization and phylogenetic tree construction using three DNA fragments (*TEF1*, *RPB1*, and *RPB2*). The results showed that potato dry rot disease in Northern Shanxi Province, is caused by five pathogenic species: *Fusarium sambucinum*, *F. solani*, *F.oxysporum*, *F. acuminatum*, and *F. dimerum*, among which *F. sambucinum* is the dominant species, accounting for 87.14% of all the strains, and distributed primarily in various potato-growing areas in the region. This study is the first to show that *F. dimerum* is a component of the pathogenic complex causing potato dry rot and is distributed primarily in the basin and hilly regions with relative frequencies of 3.45% and 13.04%, respectively. *Fusarium acuminatum* is distributed only in the plateau regions with a relative frequency of 5.56%.

## 1. Introduction

Potato is an important food and vegetable crop that serves as a staple food in China behind only wheat, rice, and maize [1]. Approximately 4.8 million hm^2^ of potatoes are grown in China, which ranks it first worldwide with respect to area and total production, at 28% and 23%, respectively. The planting area of potato spreads over all the provinces of China, with four main producing areas: a primary crop region in North China, a secondary crop region in the Central Plain region, a mixed crop region in Southwestern China, and a winter crop region in South China [2]. Among these, the North China primary crop region has the largest area, accounting for 42% of the total area. This is also the dominant producing area for seed potatoes, fresh potatoes, and processed special-purpose potatoes in China. The potato-planting areas in Shanxi Province are representative of the typical agricultural regions in North China, with over 90% of the planting area adopting the organic dry farming mode. This unique geographical environment (high altitude > 1200 m) and climatic conditions result in potatoes with excellent quality, making Shanxi a major production area for seed potatoes and commercial potatoes in China. As of 2018, the planted area of potato in Shanxi Province was approximately 200,000 hm^2^, forming three major potato industrial regions in the Yanmen Pass, Lvliang Mountain, and Taihang Mountain areas. Among these, the Yanmen Pass area is a golden region for potato production, accounting for approximately 50% of the total planted area of potato in Shanxi Province. These production areas have diverse geological morphologies and are classified into three major types, namely plateau, basin, and hilly.

In 2015, China proposed a strategy of establishing potato as a staple crop, which represents a major opportunity for the development of the potato industry. The planted area of potato and annual production have both increased in China, thereby continuously scaling up the potato industry. Postharvest storage losses of potato tubers range from 15% to 25% annually. To supply markets for fresh consumption and the starch processing industry, approximately 70% of the potato harvest requires storage for 3–6 months, making rot diseases an increasingly severe problem. However, dry rot disease of potato during storage, after harvest, is the primary storage disease in the potato-producing areas in the dry-crop growing regions of Shanxi and northwest China [3]. Potato dry rot has become a bottleneck restricting the sustainable and healthy development of the potato industry. The incidence of potato dry rot during storage is approximately 8–69% in the different growing areas in China, reaching up to 88.5% in severe cases. Crop loss due to potato dry rot ranges from 6% to 5% and can reach 60% in severe cases [4]. Dry rot not only affect the yield and quality of potato tubers, but its pathogenic agents also produce toxins and other secondary metabolites that are highly toxic to humans and animals [5]. Dry rot is caused by *Fusarium* spp. fungi and can infect a wide range of crops in the grass, eggplant, and legume families [6,7,8]. Seventeen species and five variants of dry-rot pathogens have been reported worldwide [9], and all of them have been reported in China. The pathogenic species and dominant strains show considerable variation in different potato-growing areas. The average incidence of potato dry rot in Shanxi Province is approximately 9% and can reach 25% in severe cases [10]. Wang et al. reported [10] that the potato dry-rot pathogens in Shanxi Province include *F. acuminatum*, *F. avenaceum*, *F. sambucinum*, *F. solani*, and *F. oxysporum*. Unfortunately, due to limitations associated with sampling sites and several samples, the species of dry-rot pathogens and dominant pathogens in the principal potato-producing region in the Yanmen Pass remain poorly characterized. To date, 17 species of potato dry-rot pathogens have been identified in China [10]. With the circulation of potatoes between regions and the planting of seed potatoes, there have been many changes in the populations of dry-rot pathogens. This has necessitated the systematic understanding of the species of dry-rot pathogens and dominant pathogens in the principal potato-producing region in the Yanmen Pass, to provide a theoretical basis for the scientific prevention and control of dry rot disease.

## 2. Materials and Methods

### 2.1. Pathogen Isolation and Purification

In 2020–2022, eight planting regions of the plateau (Zuoyun, Youyu), basin (Pingcheng, Tianzhen, Yanggao, Maozao), and hilly regions (Guangling, Hunyuan) of Northern Shanxi Province with continuous planting were selected. To collect potato disease samples, potato tubers with typical symptoms of disease were collected from 8 representative storage facilities. These symptomatic tubers, which displayed dark brown spots on the epidermis, shrinkage, and sunken lesions on the flesh, were subsequently packaged into self-sealing bags. Bags from different fields and harvest dates were strategically selected from key areas (e.g., vents, corners). From each bag, tubers were collected from both surface and core positions. In each storage facility, 10 to 13 samples of dry rot disease were collected, for a total of 93 potatoes. One sample was defined as an individual tuber. And these samples were transported to the laboratory at 18 °C, and the isolation of pathogenic fungi was performed within 6 h of collection.

The pathogenic fungi were isolated using tissue isolation methods [11,12]. Debris was cleared from the surface of the samples by rinsing them with sterile water. The surface of the tubers was wiped with cotton balls soaked in 75% alcohol. Tissue pieces (approximately 5 mm × 5 mm) were cut from the junctions of healthy and diseased tissues using a sterile dissecting knife, disinfected with 75% ethanol for 25 s, rinsed three times with sterile water, placed in a PDA (PDA, Hopebio, Qingdao, China) medium containing chloramphenicol (0.1 g/L), and incubated at 25 °C for 7 d.

Initial screening based on the color of the colonies and microscopic examination of the conidium morphology indicated the presence of *Fusarium*, and representative strains were screened. The PDA medium was inoculated with representative strains and cultured for 7 d. Spore suspensions (1 × 10^3^ CFU/mL) were prepared, from which single spores were isolated and incubated at 25 °C for 2 d [13]. To ensure purity, the hyphal tips of single colonies were precisely excised and transferred to a PDA medium, and cultured for 7 d. Following this, single-spore isolates were obtained and stored at 4 °C.

### 2.2. Molecular Characterization

For molecular identification, genomic DNA was extracted, and multiple gene regions were sequenced and analyzed as follows: (a) DNA extraction: mycelium was collected after 5 d of culture, and the DNA was extracted using a fungal genomic DNA extraction kit (Hangzhou Bioer Technology, Co., Ltd., Hangzhou, China). The translation elongation factor 1-a region (*TEF1*), RNA polymerase largest subunit region (*RPB1*), and RNA polymerase second largest subunit region (*RPB2*) were amplified using the primer pairs EF-1T/EF-2T, Fa/G2R, and 7cf/11ar (Table 1) [14,15]. (b) the PCR amplification procedures for *TEF1*, *RPB1*, and *RPB2* were as follows: pre-denaturation at 95 °C for 3 min, denaturation at 95 °C for 30 s, annealing for 40 s, extension at 72 °C for 60 s, 35 cycles, and finally extension at 72 °C for 10 min. The annealing temperatures of the three primer pairs are listed in Table 1. (c) expected fragment sizes: the anticipated PCR product sizes were approximately 600 bp for *TEF1*, 800 bp for *RPB1*, and 800 bp for *RPB2*. (d) software versions: the sequencing results of *TEF1*, *RPB1*, and *RPB2* were analyzed using T-BLASTn algorithm against the curated *Fusarium*-specific dataset of the FUSARIUM-ID v.3.0 database (http://isolate.fusariumdb.org/blast.php, accessed on 19 June 2025) and to standard BLASTn searches against the NCBI nucleotide collection (nr/nt) database (https://blast.ncbi.nlm.nih.gov/Blast.cgi, accessed on 13 November 2025), respectively. The PCR products were visualized by 1% agarose gel electrophoresis, and the products were recovered using a QIAquick Gel Extraction Kit (Qiagen, Hilden, Germany). The amplified products were purified and sent to Sangon Biotech Co., Ltd. (Shanghai, China) for sequencing, and the sequences were obtained and deposited in Genbank (accession numbers listed in Table 2).

The obtained sequences of various *Fusarium* species were first subjected to multiple alignment and analysis using DNAMAN 9.0 software. Subsequently, the sequences were then aligned with MAFFT and manually corrected using MEGA 12.1 software. For phylogenetic tree construction, the *TEF1*, *RPB1*, and *RPB2* gene sequences were concatenated end-to-end using PhyloSuite_v1.1.16. With *Macroconia leptosphaeriae* as the outgroup, a maximum likelihood (ML) phylogenetic tree was constructed in PAUP* v. 4.0b10 using the general time reversible model with gamma-distributed rate heterogeneity (GTR + G). Branch support was evaluated using 1000 bootstrap replicates.

### 2.3. Morphological Characterization

The PDA medium and SNA medium were inoculated with representative strains and incubated at 25 °C for 7 d, with a 12 h light/12 h dark cycle. The morphology and color of the pathogen colonies and the density of the hyphae were observed on PDA. Microscopic features, including the size, color, and morphology of the conidiophores and conidia [16], were observed with a random sample size of 100 for both macroconidia and microconidia, using an Olympus BX53F microscope on SNA.

### 2.4. Determination of Pathogenicity

The pathogenicity of the isolates was determined by injury inoculation, and eventually the pathogenicity was verified by re-isolating the post-inoculation strains in accordance with Koch’s postulates [17]. Strains cultured in PDA medium for 7 d were selected and a 1.0 × 10^5^ CFU/mL spore suspension was prepared. Qingshu No. 9 potatoes of similar size and free from disease and injury were selected; they were rinsed with water, disinfected by soaking in 0.5% sodium hypochlorite for 10 min, and air dried. A hole punch (5 mm diameter) was used to punch holes in the surface of the potato, which were inoculated with 5 μL of the spore suspension. All 70 representative strains were selected for pathogenicity detection. Each representative strain was used to inoculate 10 potato tubers, which were then placed in a sterile 25 °C incubator. The diameter of the lesions was measured 30 d after inoculation. Disease incidence was calculated using the following formula: (Number of symptomatic tubers/Total number of inoculated tubers) × 100%. It is noted that all tubers were artificially wounded to ensure successful infection, and thus, disease incidence reflects the aggressiveness of the pathogen rather than natural infection rates.

### 2.5. Data Statistics and Analysis

Data were analyzed with IBM SPSS statistics 27.0.1 by one-way ANOVA, and means were compared using Duncan’s multiple range test at a significance level of *p* = 0.05. Letters indicate significant differences (*p* = 0.05). All experiments were repeated three times.

## 3. Results

### 3.1. Characterization of Potato Dry Rot Disease Symptoms During Storage

**Figure 1:**
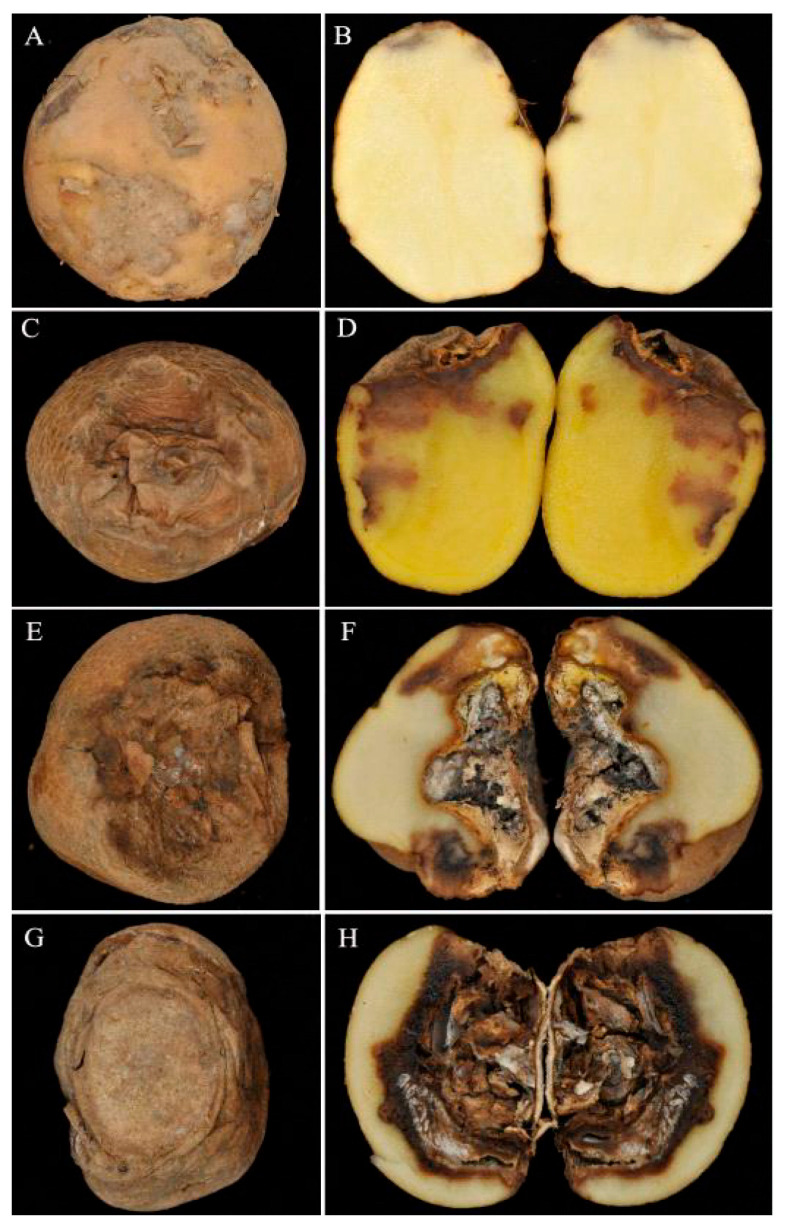
Symptoms of potato *Fusarium* dry rot during the storage period. Disease symptoms were photographed at 5 (**A**,**B**), 15 (**C**,**D**), 20 (**E**,**F**), and 30 (**G**,**H**) days post-inoculation (dpi).

In the early stages of the disease, brown, slightly depressed spots initially appeared on the skin of the potatoes, and the internal decay of the diseased potatoes exhibited a light brown coloration (Figure 1A,B). With the progression of the disease, whorls of folds appeared in the spots, and the internal decay of the diseased potatoes exhibited a dark brown coloration (Figure 1C,D). In the late stages of the disease, the surface of the potato became dry, wrinkled, and grayish-brown to dark brown. The center of the potato became hollow and filled with white hyphae (Figure 1E–H).

### 3.2. Phylogenetic Analysis

The *TEF1*, *RPB1*, and *RPB2* gene sequence were amplified from 70 representative strains isolated from the dry rot specimens from three potato-growing areas in Shanxi Province. These sequences were submitted to the GenBank database (Table 2).

**Figure 2:**
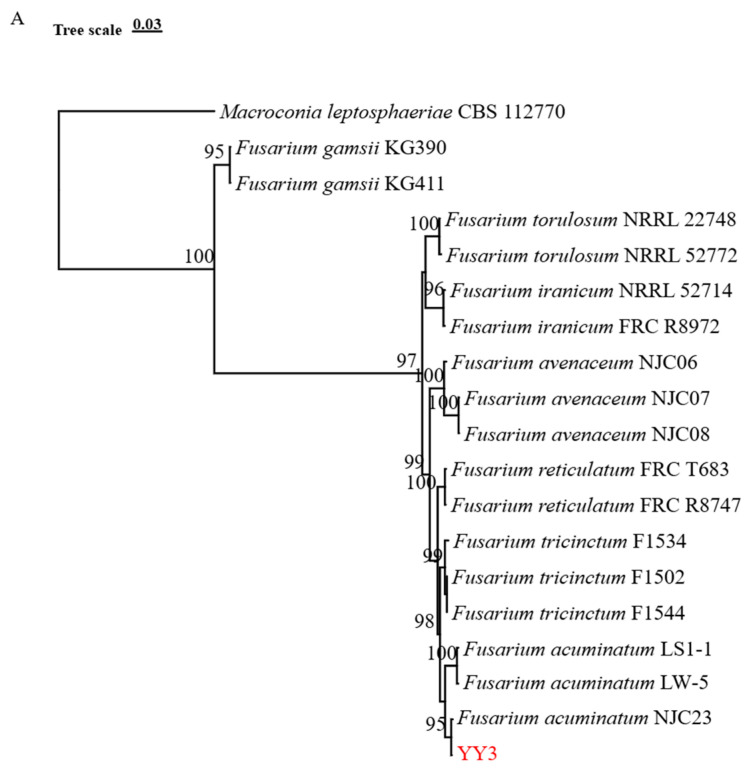

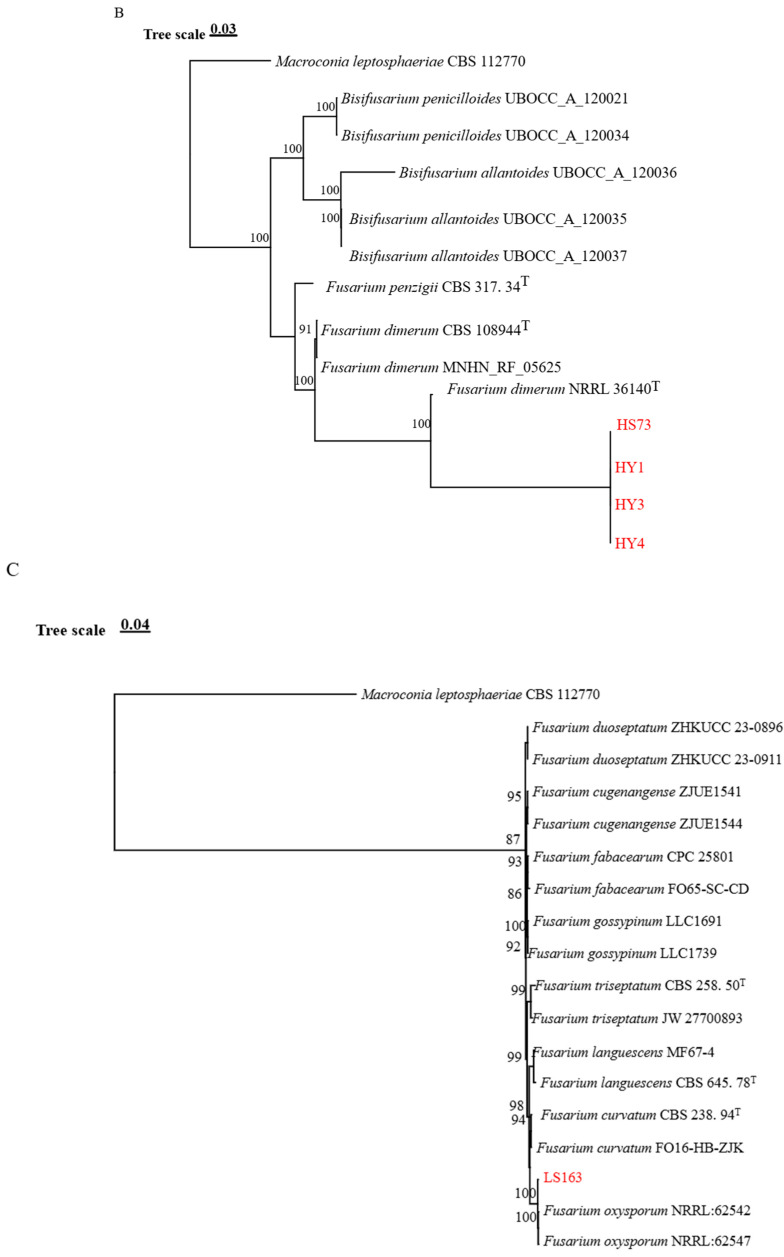

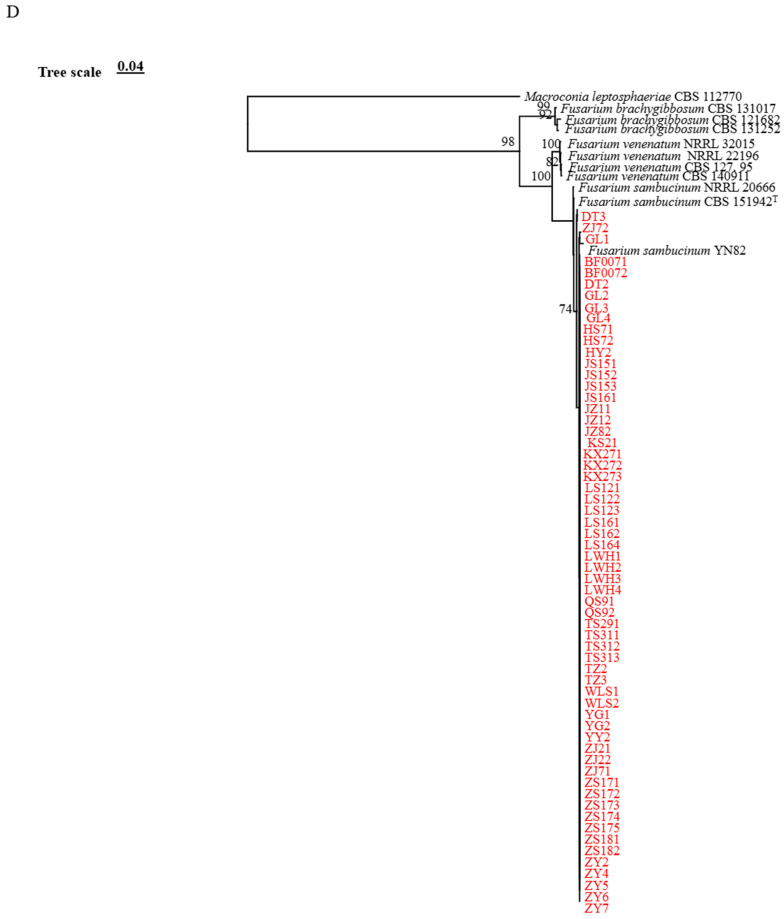

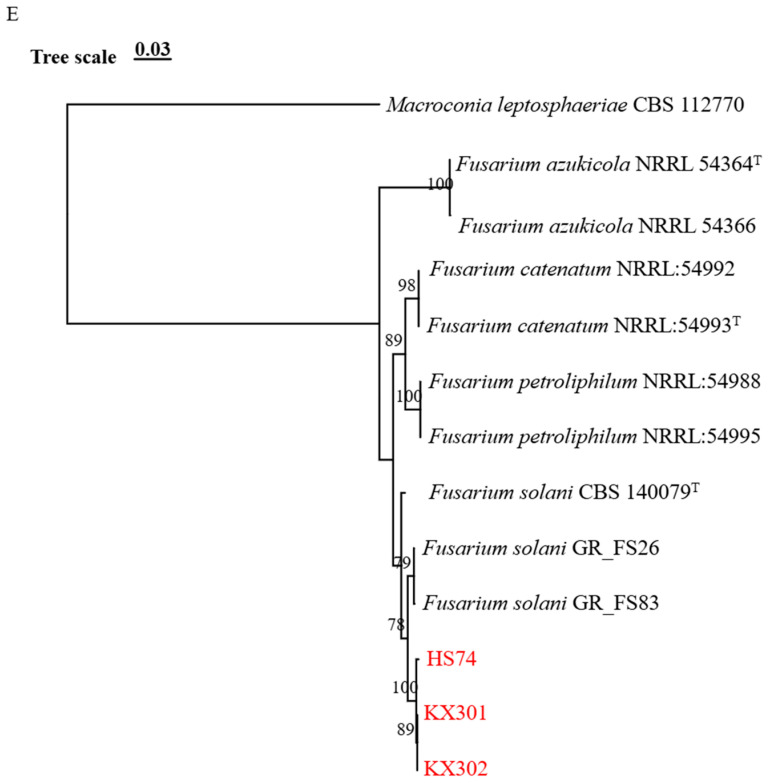
Phylogenetic tree of the *Fusarium* species isolated from potato in Shanxi Province. Maximum likelihood phylogenetic tree of *Fusarium* based on a concatenated dataset of *TEF1*, *RPB1*, and *RPB2* gene sequences. Five independent phylogenetic trees were constructed, all rooted with *Macroconia leptosphaeriae* as the outgroup. They correspond to the following five species complexes: (**A**) *F. acuminatum* to *F. tricinctum* species complex (FTSC), (**B**) *F. dimerum* to *F. dimerum* species complex (FDSC), (**C**) *F. oxysporum* to *F. oxysporum* species complex (FOSC), (**D**) *F. sambucinum* to *F. sambucinum* species complex (FSAMSC), and (**E**) *F. solani* to *F. solani* species complex (FSSC). Isolates obtained in this study are shown in red.

A phylogenetic tree was constructed using the *TEF1*, *RPB1*, and *RPB2* gene with *Macroconia leptosphaeriae* as the outgroup. The 70 screened strains clustered into five branches (Figure 2), suggesting that the potato dry-rot pathogens are classified as *F. sambucinum*, *F. solani*, *F. oxysporum*, *F. acuminatum*, and *F. dimerum*. The results showed that the 61 representative strains (BF0071, BF0072, and DT2, among others) clustered in the same branch as *F. sambucinum* with 74% bootstrap support, indicating that they are most closely related to *F. sambucinum.* Three strains (KX301, KX302, and HS74) clustered in the same branch as *F. solani* with 78% bootstrap support, indicating that they are most closely related to *F. solani*. LS163 clustered in the same branch as *F. oxysporum* with 100% bootstrap support, indicating that it is most closely related to *F. oxysporum.* YY3 clustered in the same branch as *F. acuminatum* with 95% bootstrap support, indicating that it is the most closely related to *F. acuminatum*. HY1, HY3, HY4, and HS73 clustered in the same branch as *F. dimerum* with 100% bootstrap support, indicating that they are the most closely related to *F. dimerum* (Figure 2).

### 3.3. Morphology of the Potato Dry-Rot Pathogens

**Figure 3:**
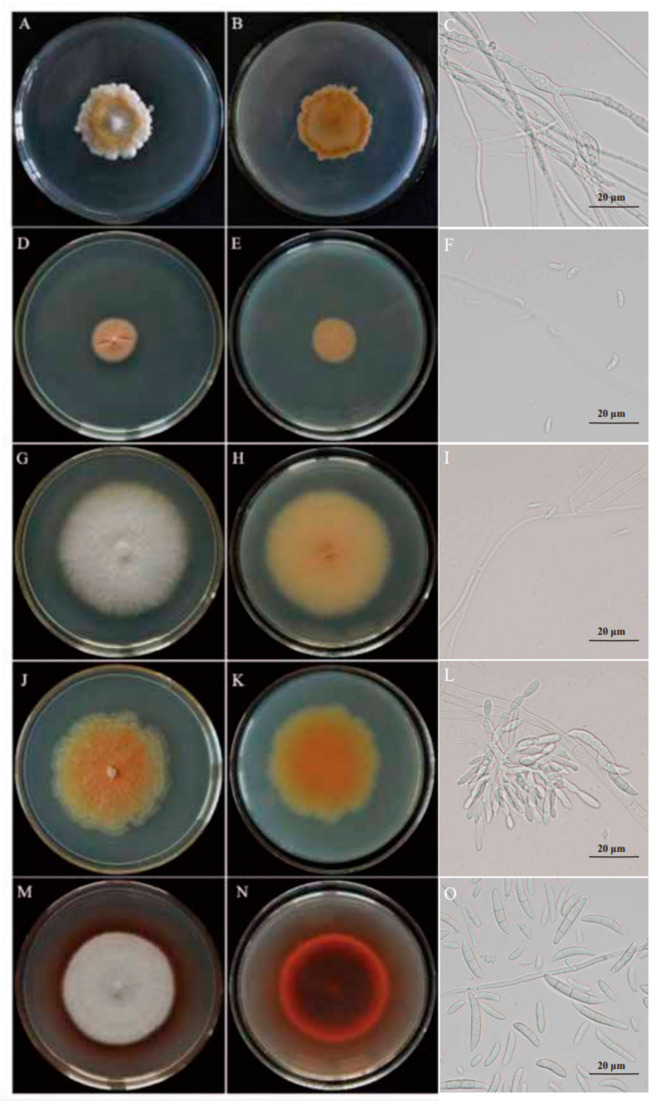
Colony Characteristics of the representative strains on the PDA plates. The characterization was carried out with strains incubated on PDA at 25 °C in the dark for 7 days (**A**,**D**,**G**,**J**,**M**), the frontal aspect of colonies on PDA medium (**B**,**E**,**H**,**K**,**N**), the reverse aspect of colonies on SNA medium (**C**,**F**,**I**,**L**,**O**), the spore morphology of colonies under an optical microscope (40×).

Based on their colony morphology, the potato dry-rot strains were broadly categorized into five major groups. On PDA medium, class 1 representative strains had white-to-light yellow, velvet-like hyphae with clear margins and colony back surfaces that were light yellow-to-yellow in color. After 7 days of incubation, the colonies attained a diameter of 3.2–4.1 cm (Figure 3A,B). Microconidia were ovel-ellipsoidal, and 3.2–4.5 μm × 2.2–3.7 μm in size. Macroconidia were rarely observed. (Figure 3C). Class 2 representative strains had white, velvet-like hyphae with clear margins and colony back surfaces that were light yellow in color. After 7 days of incubation, the colonies attained a diameter of 1.6–2.2 cm (Figure 3D,E). Microconidia were crescent-shaped to ovoid, occasionally with pointed apices, and 4.8–7.3 μm × 2.3–3.8 μm in size. Macroconidia were not produced (Figure 3F). Class 3 representative strains had white, velvet-like hyphae with clear margins and colony back surfaces that were light yellow in color. After 7 days of incubation, the colonies attained a diameter of 5.3–6.2 cm (Figure 3G,H). The conidia (microconidia and macroconidia) were elliptical with tapered ends, and 3.2–6.9 μm × 2.2–3.4 μm in size (Figure 3I). Class 4 representative strains had white-to-grayish-white, cotton-to-velvet-like hyphae that were well developed in the center and diffused at the margins. The colony back surfaces were dark yellow and could produce a purple pigment. After 7 days of incubation, the colonies attained a diameter of 4.5–5.3 cm (Figure 3J,K). The fungus produced both macroconidia and microconidia. Macroconidia were falcate and 2 to 4 septate. Microconidia were fusiform, 0 to 1 septate and 7.6–35.7 μm × 2.1–3.7 μm in size. Chlamydospores were formed in clusters or chains (Figure 3L). Class 5 representative strains had white-to-grayish-white, cotton-to-velvet-like hyphae that were well developed in the center and had clear margins. The colony back surfaces were dark red and could produce a red pigment. After 7 days of incubation, the colonies attained a diameter of 5.1–5.5 cm (Figure 3M,N). Macroconidia were falcate and 2 to 3 septate, while the microconidia were elliptical to ovoid and 1 to 2 septate. Conidia (microconidia and macroconidia) measured 4.3–36.2 μm × 2.2–3.6 μm (Figure 3O).

### 3.4. Pathogenicity Analysis

**Figure 4:**
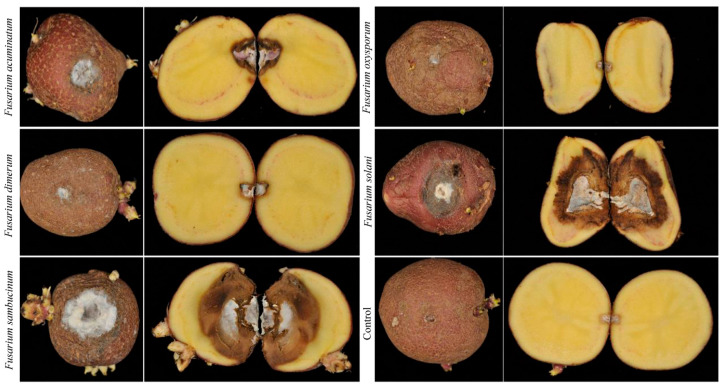
Pathogenicity of *Fusarium acuminatum*, *F. dimerum*, *F. sambucinum*, *F. oxysporum*, and *F. solani*. Representative isolates of *Fusarium* species inoculated on potato tubers (Qingshu No. 9). Species and corresponding strain numbers are as follows: *F. acuminatum* (YY3), *F. dimerum* (HY3), *F. sambucinum* (LWH1), *F. oxysporum* (LS163), and *F. solani* (KX301). All tubers were incubated at 25 °C for 30 d.

**Figure 5:**
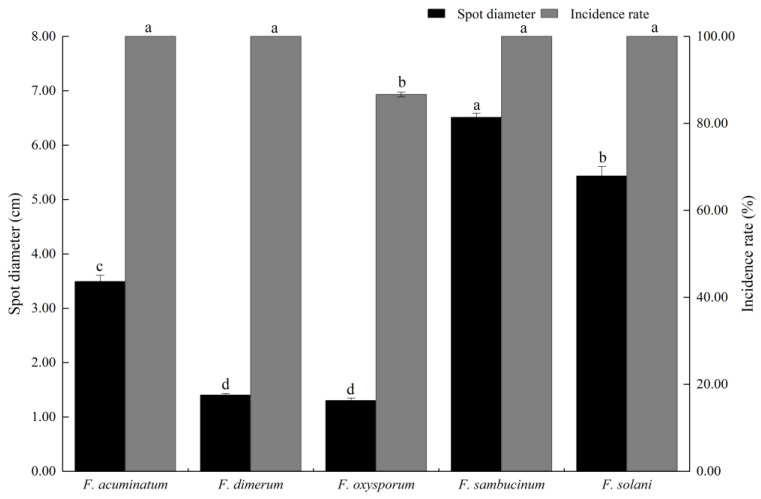
Spot diameter and incidence rate for each *Fusarium* species (70 strains). *Fusarium acuminatum* (n = 1), *F. dimerum* (n = 4), *F. sambucinum* (n = 61), *F. oxysporum* (n = 1), and *F. solani* (n = 3). Different letters represent significant differences in spot diameter among various *Fusarium* species (*p* < 0.05). The error bars represent the mean ± 95% Cl.

After inoculation with *F. acuminatum*, *F. dimerum*, *F. sambucinum*, *F. oxysporum*, and *F. solani*, the symptoms in the healthy potatoes were generally consistent with those in the tuber bulking stage, and the *Fusarium* species isolated from each diseased plant was identical to those used during inoculation, indicating that *F. acuminatum*, *F. dimerum*, *F. sambucinum*, *F. oxysporum*, and *F. solani* were the pathogenic fungi responsible for the potato dry rot during storage. The pathogenicity of the five *Fusarium* species varied significantly, with large differences in the diameter of the spots on the potatoes. *Fusarium sambucinum* was the most pathogenic, with an incidence of 100% and a spot diameter of 6.51 ± 0.08 cm, which was significantly higher than that of the other four *Fusarium* species (*p* < 0.05). *Fusarium solani* was the next most pathogenic, with an incidence of 100% and a spot diameter of 5.43 ± 0.18 cm, exhibiting a significant difference compared to the other three *Fusarium* species. *Fusarium acuminatum* was less pathogenic, with an incidence and spot diameter of 100% and 3.49 ± 0.12 cm, respectively. The incidence and spot diameter of *F. dimerum* were 100% and 1.40 ± 0.03 cm, respectively. *Fusarium oxysporum* was the least pathogenic, with an incidence and spot diameter of 86.67% and 1.30 ± 0.20 cm, respectively (Figure 4 and Figure 5).

Pathogenicity analysis revealed a clear virulence gradient among the five *Fusarium* species. *F. sambucinum* and *F. solani* constituted a highly aggressive group, causing the most extensive lesions. In contrast, *F. acuminatum*, *F. dimerum*, and *F. oxysporum* formed a low-virulence group, with the latter also exhibiting reduced infectivity. Notably, the isolates of *F. oxysporum* and *F. dimerum* caused minimal tissue damage, suggesting that they might not be primary causal agents of dry rot but rather secondary colonizers that invade tissue following establishment by more aggressive microbes.

### 3.5. Composition and Distribution of the Pathogens Causing Potato Dry Rot in Three Potato-Growing Areas

**Figure 6:**
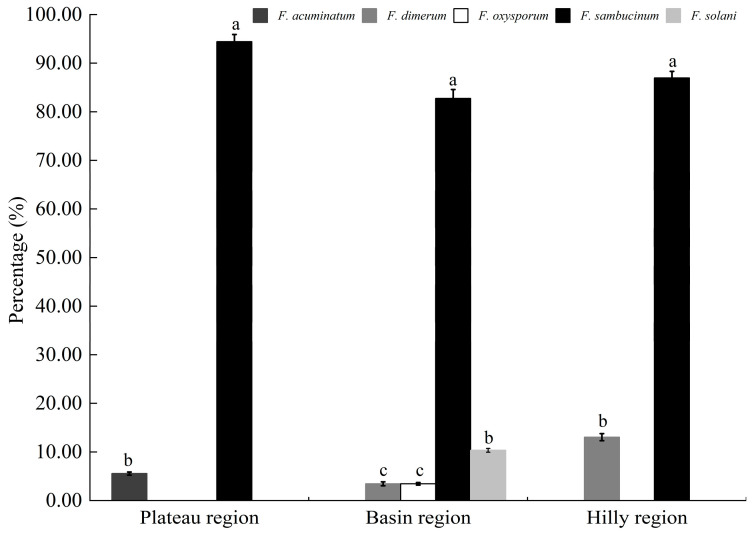
Distribution and species composition of the *Fusarium* spp. from potato-planting areas in Shanxi Province. Different lowercase letters in the figure indicated that there were significant differences among different *Fusarium* species in different regions (*p* < 0.05).

The pathogens causing potato dry rot in Shanxi Province belonged to five pathogenic species. Among them, *F*. *sambucinum* was distributed in three potato-growing areas of Shanxi Province. Notably, *F*. *sambucinum* accounted for 87.14% of the total number of strains. Only two pathogenic species, *F. acuminatum* and *F. sambucinum*, were isolated from the plateau region. Four pathogenic species were isolated from the basin region, which were *F. dimerum*, *F. oxysporum*, *F. sambucinum*, and *F. solani*. Additionally, *F. dimerum* and *F. sambucinum* were distributed in the hilly region (Figure 6).

## 4. Discussion

Dry rot is a major disease experienced in potatoes during storage. Understanding the composition and distribution of dry rot fungus populations provides a scientific basis for targeted prevention and the control of potato dry rot. Potato dry-rot pathogens in various regions worldwide are complex and diverse, with approximately 17 species in China [12,18,19,20], including *F. acuminatum*, *F. avenaceum*, *F. culmorum*, *F. equiseti*, *F. gibbosum*, *F. macroceras*, *F. moniliforme*, *F. redolens*, *F. sambucinum*, *F. semitectum*, *F. solani*, *F. sporotrichiodes*, *F. sulphureum*, *F. trichothecioides*, and *F. tricinctum*, of which the 5 dominant species in the North China primary crop region are *F. avenaceum*, *F. acuminatum*, *F. sambucinum*, *F. solani*, and *F. sporotrichiodes*. Wang et al. reported [10] that among the five dominant pathogenic species in the potato-growing areas of Shanxi Province did not include *F. sporotrichiodes*. but *F. oxysporum* was. Potato dry rot has also been reported in other countries, including the United Kingdom, South Africa, the mid-northern United States, and Egypt [21,22,23,24,25]. The dominant species of potato dry rot disease across various regions of the world primarily concentrate on *F. sambucinum*, *F. oxysporum*, and *F. solani*, with *F. sambucinum* presenting the utmost pathogenicity and *F. oxysporum* the widest infective capacity [26,27,28,29,30,31,32,33,34]. In the present study, we found that the pathogenic agents of dry rot disease in the principal potato-producing areas in the Yanmen Pass included *F. sambucinum*, *F. solani*, *F. oxysporum*, *F. acuminatum*, and *F. dimerum*, of which *F. sambucinum* and *F. solani* accounted for large proportions and were the dominant species. However, *F. avenaceum* was not isolated from the Yanmen Pass potato-producing area, indicating that the composition of pathogenic species has changed over time and in response to changes in planting structure. Potato dry-rot pathogens are complex and diverse in different growing regions. It has been hypothesized that this is attributed to differences in planting structures, among other reasons. In Gansu Province, characterized by a temperate arid continental climate, *F. solani* and *F. oxysporum* have been reported as the predominant species [35]. In contrast, our study, conducted across three distinct regions in northern Shanxi Province, identified *F. sambucinum* as the dominant pathogen. This shift in species dominance may be attributed to the distinct climatic, geographical, and soil conditions specific to northern Shanxi. In addition, comprehensive and systematic studies on dry-rot pathogen species in various potato-producing areas remain essential.

The present study is the first to identify *F. dimerum* as a fungal species associated with potato dry rot disease, accounting for 5.71% of all strains. In China, *F. dimerum* primarily causes diseases in bananas, dragon fruit, broad beans, and peas. Banana and dragon fruit are primarily distributed in South China and cannot be cultivated in Shanxi Province. However, broad beans and peas are among the major small grain crops traditionally cultivated in Shanxi Province and are also common crops in the predominantly potato-growing area of the Yanmen Pass. Belete E et al. reported that broad bean root rot is caused by *Fusarium* fungi [36]. The pathogens of pea root rot include co-infection by over 10 species of *Fusarium* and *Pythium* [37], among which the major pathogens include *F. solani*, *F. oxysporum*, *F. dimerum*, and *Rhizoctonia solani*. *Fusarium solani*, *F. oxysporum*, *F. acuminatum*, and *F. dimerum* are the primary pathogens of broad bean and pea root rot and are generally the same pathogens as the dry-rot pathogens in the Northern Shanxi potato-producing area. There is a tradition of crop rotation between potatoes, broad beans, and peas in the Yanmen Pass potato-producing area. It has been hypothesized that this might have led to increased *F. dimerum* abundance in potato following broad bean and pea crop rotation. The principal source of potato dry-rot pathogens is the soil, and these pathogens can overwinter and survive for long periods [38]. Crop rotation is an important measure for controlling potato dry rot and is effective in reducing *Fusarium* levels in the soil. Potato is a member of the nightshade family of crops, and crop rotation with other nightshades, such as tomato, tobacco, eggplant, and pepper, as well as other tuberous crops such as sweet potato, should be avoided [39,40]. Peters et al. found that the incidence of potato dry rot was reduced when potato was rotated with alfalfa, clover, and cereals [41]. Therefore, selective crop rotation with non-*Fusarium* host crops may serve as a potential strategy for controlling potato dry rot.

This study revealed that *F. sambucinum*, *F. solani*, *F. oxysporum*, *F. acuminatum*, and *F. dimerum* could not directly invade potatoes through the epidermis but instead required the presence of wounds. Potatoes can be susceptible to *Fusarium* infection through injuries; therefore, caution must be exercised to keep potatoes intact during sowing, harvesting, and transport to prevent or minimize mechanical damage. In addition, the timely elimination of damaged potatoes is essential for the prevention and control of potato dry rot disease during storage. Agricultural control is only one of many methods of controlling potato dry rot, demonstrating its ineffectiveness as a standalone solution for disease control. Disease prevention and control rely on accurate identification of the dominant species and population structure of dry rot in the different potato-producing areas. Future selection of effective potato dry rot prevention and control measures should be conducted in accordance with the different dominant species and population structure, taking into account the local conditions.

## 5. Conclusions

In this research, to clarify the species, distribution, and potential pathogenicity of pathogens associated with potato dry rot in the northern part of Shanxi Province, we collected 93 samples showing symptoms of dry rot and isolated 70 pathogenic strains. By analyzing the cultural characteristics and conducting phylogenetic analysis of the strains we successfully identified five distinct *Fusarium* species. Pathogenicity analysis confirmed that *F. sambucinum*, *F. solani*, *F. oxysporum*, *F. acuminatum*, and *F. dimerum* are the causal agents of potato dry rot during the storage in three major potato-growing regions of northern part of Shanxi Province. Among these species, *F. sambucinum* exhibits the strongest pathogenicity and the widest distribution (occurring in all three regions areas), making it the dominant species. Furthermore, this study is the first to identify that *F*. *dimerum* is associated with potato dry rot and can act as a causal agent of the disease. However, its pathogenicity is significantly lower than that of other species, suggesting it primarily functions as a weak pathogen or secondary colonizer. The findings of this research provide a scientific basis for the comprehensive prevention and control of potato dry rot in the northern part of Shanxi Province.

## Figures and Tables

**Table 1:** Primer pairs and annealing temperatures used in the study.

Amplified Region	Primer Name	Sequence (5′-3′)	Annealing Temperatures (°C)
*TEF1*	EF-1T	ATGGGTAAGGAAGACAAGAC	57.5
	EF-2T	GGAAGTACCAGTGATCATGTT	
*RPB1*	Fa	CAYAARGARTCYATGATGGGWC	55
	G2R	GTCATYTGDGTDGCDGGYTCDCC	
*RPB2*	7cf	ATGGGYAARCAAGCYATGGG	55
	11ar	GCRTGGATCTTRTCRTCSACC	

**Table 2:** Species, Strain numbers, Host, and GenBank accession numbers of the taxa used for phylogenetic analyses. Isolates obtained in this study are shown in bold. ^T^ = Ex-type strains.

Species	Strain No.	Host	GenBank Accession No.		
			*TEF1*	*RPB1*	*RPB2*
*F. acuminatum*	**YY3**	*S. tuberosum*	PV382882	PV854763	PV854833
	NJC23	*Allium sativum*	OL741722	OL741716	OL741720
	LW-5	-	OP838084	OP838085	OP838086
	LS1-1	Wheat	OP105209	OP785217	OP785270
*F. gamsii*	KG411	*Triticum aestivum*	ON960708	ON960644	ON960692
	KG390	*Triticum aestivum*	ON960707	ON960643	ON960691
*F. torulosum*	NRRL 22748	-	OL772877	OL773029	OL773181
	NRRL 52772	-	OL772887	OL773039	OL773191
*F. iranicum*	NRRL 52714	-	OL772883	OL773035	OL773187
	FRC R8972	-	OL772864	OL773016	OL773168
*F. avenaceum*	NJC06	Kiwi tree	OL439731	OL439737	OL439740
	NJC07	Kiwi tree	OL439732	OL439738	OL439741
	NJC08	Kiwi tree	OL439733	OL439739	OL439742
*F. reticulatum*	FRC T683	-	OL772860	OL773012	OL773164
	FRC R8747	-	OL772859	OL773011	OL773163
*F. tricinctum*	F1502	-	OL964794	OL658681	OL658756
	F1534	-	OL964796	OL658676	OL658761
	F1544	-	OL964791	OL658669	OL658768
*F. dimerum*	**HS73**	*S. tuberosum*	PV382890	PV854767	PV854837
	**HY1**	*S. tuberosum*	PV382889	PV854766	PV854836
	**HY3**	*S. tuberosum*	PV382888	PV854765	PV854835
	**HY4**	*S. tuberosum*	PV382887	PV854764	PV854834
	MNHN_RF_05625	-	MW811085	MW811058	MW811070
	NRRL 36140 ^T^	-	HM347133	HM347203	HM347218
	CBS 108944 ^T^	-	KR673912	KM232212	KR674020
*F. penzigii*	CBS 317.34 ^T^	-	EU926324	KM232211	KM232362
*B* *. allantoides*	UBOCC_A_120037	-	MW811088	MW811055	MW811073
	UBOCC_A_120036	-	MW811087	MW811087	MW811072
	UBOCC_A_120035	-	MW811075	MW811046	MW811060
*B* *. penicilloides*	UBOCC_A_120021	-	MW811081	MW811051	MW811066
	UBOCC_A_120034	-	MW811080	MW811050	MW811065
*F. oxysporum*	**LS163**	*S. tuberosum*	PV382883	PV854762	PV854832
	NRRL:62542	-	KC808229	KC808302	KC808365
	NRRL:62547	-	KC808224	KC808304	KC808367
*F. triseptatum*	CBS 258.50 ^T^	*Ipomoea batatas*	MH484964	MW928820	MH484873
	JW 277008	-	MZ921888	MZ921662	MZ921757
*F. languescens*	CBS 645.78 ^T^	*S. lycopersicum*	MH484971	MW928813	MH484880
	MF67-4	*Dioscorea esculent* *a*	PQ774592	PQ774580	PQ774586
*F. curvatum*	CBS 238.94 ^T^	*Beaucarnea* sp.	MH484984	MW928804	MH484893
	FO16-HB-ZJK	-	PQ300823	PQ296597	PQ296676
*F. duoseptatum*	ZHKUCC 23-0911	*Strelitzia reginae*	PQ316056	PQ468017	PQ356493
	ZHKUCC 23-0896	*Strelitzia reginae*	PQ316055	PQ468016	PQ356492
*F. fabacearum*	CPC 25801	-	MH485029	MZ921691	MH484938
	FO65-SC-CD	-	PQ300866	PQ296640	PQ296719
*F. gossypinum*	LLC1739	Soil	OP487221	OP486374	OP486789
	LLC1691	Soil	OP487220	OP486373	OP486788
*F. cugenangense*	ZJUE1544	*Citrus unshiu*	PX130564	PV983890	PX130492
	ZJUE1541	*Citrus unshiu*	PX130563	PV983889	PX130491
*F. sambucinum*	**BF0071**	*S. tuberosum*	PV382910	PV854758	PV854828
	**BF0072**	*S. tuberosum*	PV382892	PV854757	PV854827
	CBS 151942 ^T^	*Sambucus nigra*	PQ260927	PQ280949	PQ274212
	**DT2**	*S. tuberosum*	PV382943	PV854756	PV854826
	**DT3**	*S. tuberosum*	PV382891	PV854755	PV854825
	**GL1**	*S. tuberosum*	PV382948	PV854754	PV854824
	**GL2**	*S. tuberosum*	PV382947	PV854753	PV854823
	**GL3**	*S. tuberosum*	PV382934	PV854752	PV854822
	**GL4**	*S. tuberosum*	PV382924	PV854751	PV854821
	**HS71**	*S. tuberosum*	PV382904	PV854750	PV854820
	**HS72**	*S. tuberosum*	PV382927	PV854749	PV854819
	**HY2**	*S. tuberosum*	PV382951	PV854748	PV854818
	**JS151**	*S. tuberosum*	PV382906	PV854747	PV854817
	**JS152**	*S. tuberosum*	PV382919	PV854746	PV854816
	**JS153**	*S. tuberosum*	PV382937	PV854745	PV854815
	**JS161**	*S. tuberosum*	PV382905	PV854744	PV854814
	**JZ11**	*S. tuberosum*	PV382908	PV854743	PV854813
	**JZ12**	*S. tuberosum*	PV382939	PV854742	PV854812
	**JZ82**	*S. tuberosum*	PV382907	PV854741	PV854811
	**KS21**	*S. tuberosum*	PV382901	PV854740	PV854810
	**KX271**	*S. tuberosum*	PV382917	PV854739	PV854809
	**KX272**	*S. tuberosum*	PV382945	PV854738	PV854808
	**KX273**	*S. tuberosum*	PV382921	PV854737	PV854807
	**LS121**	*S. tuberosum*	PV382946	PV854736	PV854806
	**LS122**	*S. tuberosum*	PV382930	PV854735	PV854805
	**LS123**	*S. tuberosum*	PV382914	PV854734	PV854804
	**LS161**	*S. tuberosum*	PV382920	PV854733	PV854803
	**LS162**	*S. tuberosum*	PV382893	PV854732	PV854802
	**LS164**	*S. tuberosum*	PV382896	PV854731	PV854801
	**LWH1**	*S. tuberosum*	PV382944	PV854730	PV854800
	**LWH2**	*S. tuberosum*	PV382909	PV854729	PV854799
	**LWH3**	*S. tuberosum*	PV382923	PV854728	PV854798
	**LWH4**	*S. tuberosum*	PV382895	PV854727	PV854797
	NRRL 20666	-	MW233072	MW233243	MW233415
	**QS91**	*S. tuberosum*	PV382936	PV854726	PV854796
	**QS92**	*S. tuberosum*	PV382913	PV854725	PV854795
	**TS291**	*S. tuberosum*	PV382929	PV854724	PV854794
	**TS311**	*S. tuberosum*	PV382935	PV854723	PV854793
	**TS312**	*S. tuberosum*	PV382940	PV854722	PV854792
	**TS313**	*S. tuberosum*	PV382915	PV854721	PV854791
	**TZ2**	*S. tuberosum*	PV382903	PV854720	PV854790
	**TZ3**	*S. tuberosum*	PV382911	PV854719	PV854789
	**WLS1**	*S. tuberosum*	PV382942	PV854718	PV854788
	**WLS2**	*S. tuberosum*	PV382894	PV854717	PV854787
	**YG1**	*S. tuberosum*	PV382898	PV854716	PV854786
	**YG2**	*S. tuberosum*	PV382899	PV854715	PV854785
	YN82	-	OR019814	OR019820	OR019826
	**YY2**	*S. tuberosum*	PV382932	PV854714	PV854784
	**ZJ21**	*S. tuberosum*	PV382916	PV854713	PV854783
	**ZJ22**	*S. tuberosum*	PV382925	PV854712	PV854782
	**ZJ71**	*S. tuberosum*	PV382931	PV854711	PV854781
	**ZJ72**	*S. tuberosum*	PV382928	PV854710	PV854780
	**ZS171**	*S. tuberosum*	PV382897	PV854709	PV854779
	**ZS172**	*S. tuberosum*	PV382938	PV854708	PV854778
	**ZS173**	*S. tuberosum*	PV382912	PV854707	PV854777
	**ZS174**	*S. tuberosum*	PV382922	PV854706	PV854776
	**ZS175**	*S. tuberosum*	PV382941	PV854705	PV854775
	**ZS181**	*S. tuberosum*	PV382933	PV854704	PV854774
	**ZS182**	*S. tuberosum*	PV382918	PV854703	PV854773
	**ZY2**	*S. tuberosum*	PV382949	PV854702	PV854772
	**ZY4**	*S. tuberosum*	PV382950	PV854701	PV854771
	**ZY5**	*S. tuberosum*	PV382926	PV854700	PV854770
	**ZY6**	*S. tuberosum*	PV382900	PV854699	PV854769
	**ZY7**	*S. tuberosum*	PV382902	PV854698	PV854768
*F. brachygibbosum*	CBS 121682	Stone	PQ260819	PQ280843	PQ274106
	CBS 131017	*Agropyron* sp.	PQ260820	PQ280844	PQ274107
	CBS 131252	*Triticum* sp.	PQ260821	PQ280845	PQ274108
*F. venenatum*	NRRL 32015	-	MW233109	MW233281	MW233453
	NRRL 22196	-	MW233078	MW233249	MW233421
	CBS 140911	Grass	PQ260954	PQ280984	PQ274241
	CBS 127.95	*S. tuberosum*	PQ260953	PQ280983	PQ274240
*F. solani*	**HS74**	*S. tuberosum*	PV382886	PV854761	PV854831
	**KX301**	*S. tuberosum*	PV382884	PV854759	PV854829
	**KX302**	*S. tuberosum*	PV382885	PV854760	PV854830
	CBS 102429	Bark	KM231936	KM232227	KM232376
	GR_FS26	Asparagus root	MT305228	MT305111	MT305169
	GR_FS83	Asparagus root	MT305232	MT305115	MT305173
*F. azukicola*	NRRL 54364 ^T^	-	JQ670137	KJ511276	KJ511287
	NRRL 54366	-	JQ670139	KJ511277	KJ511288
*F. catenatum*	NRRL:54993 ^T^	-	KC808214	KC808292	KC808355
	NRRL:54992	-	KC808213	KC808291	KC808354
*F. petroliphilum*	NRRL:54995	-	KC808215	KC808293	KC808356
*M* *. leptosphaeriae*	CBS 112770	*Cucurbitaria laburni*	KM231960	KM232256	KM232389

## Data Availability

The original contributions presented in this study are included in the article. Further inquiries can be directed to the corresponding author.
